# Comparative transcriptomic and lipidomic analyses indicate that cold stress enhanced the production of the long C18–C22 polyunsaturated fatty acids in *Aurantiochytrium* sp.

**DOI:** 10.3389/fmicb.2022.915773

**Published:** 2022-09-20

**Authors:** Yingjie Song, Zhangli Hu, Zheng Xiong, Shuangfei Li, Wei Liu, Tian Tian, Xuewei Yang

**Affiliations:** ^1^Guangdong Technology Research Center for Marine Algal Bioengineering, Guangdong Key Laboratory of Plant Epigenetics, Shenzhen Engineering Laboratory for Marine Algal Biotechnology, Longhua Innovation Institute for Biotechnology, College of Life Sciences and Oceanography, Shenzhen University, Shenzhen, China; ^2^College of Physics and Optoelectronic Engineering, Shenzhen University, Shenzhen, China; ^3^Shenzhen Key Laboratory of Marine Biological Resources and Ecology Environment, Shenzhen Key Laboratory of Microbial Genetic Engineering, College of Life Sciences and Oceanography, Shenzhen University, Shenzhen, China; ^4^Shenzhen Institute of Modern Agricultural Equipment, Shenzhen, China; ^5^State Key Laboratory of Synthetic Chemistry, Department of Chemistry, The University of Hong Kong, Pokfulam, Hong Kong SAR, China

**Keywords:** *Aurantiochytrium* sp., fatty acids, biosynthesis, lipidomics, transcriptomics

## Abstract

*Aurantiochytrium* sp. belonging to Thraustochytrids are known for their capacity to produce long-chain polyunsaturated fatty acids (PUFAs). However, effects of cold stress accompanied with staged-temperature control on the fatty acid metabolism in *Aurantiochytrium* sp. were rarely studied. In this study, cold stress (15°C, 5°C) was applied for *Aurantiochytrium* sp., with the physiological responses (morphology, growth, fatty acid profiling) and gene expression related FA synthesis, lipid metabolism, and regulatory processes was observed. Results showed that there is a significant change for the lipid types under 5°C (251 species) and 15°C (97 species) treatment. The 5°C treatment was benefit for the C18–C22 PUFAs with the yield of docosahexaenoic acid (DHA) increased to 1.25 times. After incubation at 15°C, the accumulation of eicosadienoic acid (EA) (20:2) was increased to 2.00-fold. Based on transcriptomic and qPCR analysis, an increase in genes involved in fatty acid synthase (FAS) and polyketide synthase (PKS) pathways was observed under low-temperature treatment. With upregulation of 3-ketoacyl-CoA synthase (2.44-fold), ketoreductase (2.50-fold), and dTDP-glucose 4,6-Dehydratase (rfbB) (2.31-fold) involved in PKS pathway, the accumulation of DHA was enhanced under 5°C. While, FAS and fatty elongase 3 (ELO) involved in the FAS pathway were upregulated (1.55-fold and 2.45-fold, respectively) to accumulate PUFAs at 15°C. Additionally, glycerol-3-phosphate acyltransferase (GPAT), lysophospholipid acyltransferase (LPAT), phosphatidic acid phosphatase (PAP), phosphatidylserine synthase (PSS), and phosphatidylserine decarboxylase (PSD) involved in glycerophospholipid biosynthesis were upregulated at 5°C increasing the accumulation of phosphatidic acid (PA), phosphatidylcholine (PC), phosphatidylethanolamine (PE), phosphatidylglycerol (PG), and phosphatidylinositol (PI). However, glycolysis and the TCA cycle were inhibited under 5°C. This study provides a contribution to the application of two-staged temperature control in the *Aurantiochytrium* sp. fermentation for producing cold stress-enhancing PUFAs, in order to better understand the function of the key genes for future genetic engineering.

## Graphical Abstract

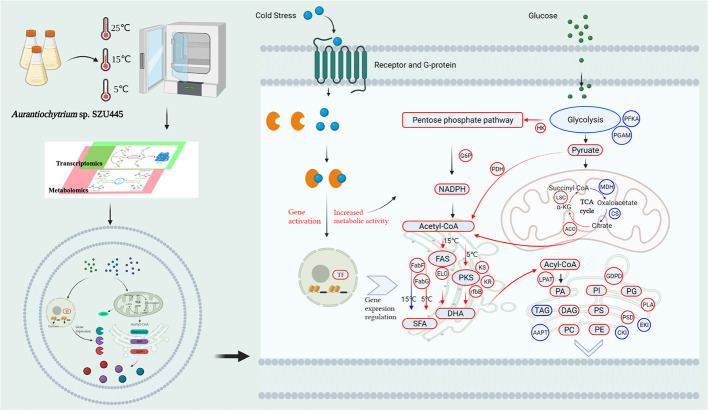

## Introduction

*Aurantiochytrium* sp. belongs to Thraustochytrids (Naganuma et al., 1998; Marchan et al., 2018), which is well known for its capacity to accumulate an abundance of fatty acids (FAs), especially very long-chain polyunsaturated fatty acids (VLC-PUFAs) (Cheng et al., 2016; Cui et al., 2016). *Aurantiochytrium* sp. plays a significant role as the food source for picoplankton and marine microbial feeders, and has been established as the candidate for polyunsaturated fatty acids (PUFAs) production (Ju et al., 2019; Ji and Ledesma-Amaro, 2020). PUFAs in *Aurantiochytrium* sp. mainly include docosahexaenoic acid (DHA) (C22:6, n-3), docosapentaenoic acid (DPA) (C22:5, n-6), and eicosapentaenoic acid (EPA) (C20:5, n-3). Some *Aurantiochytrium* sp. strains have been used for the industrial production of DHA, such as *Aurantiochytrium* sp. SD116 (Wang et al., 2020). The fermentation conditions have been studied to increase the production of PUFAs in *Aurantiochytrium* sp., such as supplying the nitrogen starvation to induce an improvement in DHA yield through PUFA-synthase genes’ upregulation (Heggeset et al., 2019). Moreover, low temperatures have been used to improve production in some microorganisms, such as *Pavlova lutheri* (Guiheneuf and Stengel, 2017) and *Chlamydomonas malina* (Morales-Sanchez et al., 2020).

The low temperature was considered as critical factor that affects FAs yield and composition in microorganisms, which has been used to improve lipid production in some microorganisms. Examples of them are *Auxenochlorella protothecoides* (Xing et al., 2018), *Aurantiochytrium* sp. (Wang et al., 2022), *Chlorella* sp. (Zheng et al., 2021), *Cylindrotheca closterium* (Almeyda et al., 2020), *Isochrysis galbana* (Cao et al., 2020), *Nannochloropsis salina* (Gill et al., 2018), and *Scenedesmus* sp. (Calhoun et al., 2021) to investigate its effects on the yield of fatty acids (summarized as Supplementary Table 1). Low temperature such as 5°C was also used to stimulate lipids synthesis in various algae and microorganism (Lu et al., 2009; Sirisuk et al., 2018; Qin et al., 2021). In *Thraustochytriidae* sp. Z105, low temperature enhanced lipid, DHA and biomass accumulation (Zhou et al., 2010). In *Aurantiochytrium* sp., a substantial number of genes have been found, which have potential roles in the adaptation to low temperature, and influence lipid composition, such as lysophosphatidic acid (LPA) acyltransferase, PUFA synthases, and long-chain acyl-CoA synthases (Ma et al., 2015, 2017). However, most of the studies focused on the one-step fermentation with low temperature (shown as Supplementary Table 1). It was reported that compares with one-step fermentation, the staged temperature control method is important for accumulating the total amount of lipids (Almeyda et al., 2020). Staged temperature control fermentation strategy consists of two main processes with various temperatures. The first process is applied with optimal cell growth temperature which is a benefit for the cell growth in the logarithmic growth stage to obtain a high yield of cells (Gill et al., 2018; Almeyda et al., 2020). The second process is applied with stress condition such as low temperature to stimulate the accumulation of the secondary metabolites. For instance, moving *Cylindrotheca closterium* from 20°C incubator (stationary phase) to 10°C, enhanced the accumulation of PUFA (Almeyda et al., 2020). The staged temperature control method combined with cold stress stimulation strategy which is important for fatty acids accumulation has been studied in the previous research. However, it is rarely studied in *Aurantiochytrium* sp.

Lipidomic is a novel subject that studies the mechanism of action of lipid metabolic regulation in various biological processes (Kind et al., 2013). Recently, lipidomic has been widely used in plant fields. For example, the identification of 283 species of lipid provided insights into lipid metabolism in green tea manufacturing and its role in green tea sensory quality (Li et al., 2021). It was also applied to detect the triacylglycerol (TAG) and phosphatidylcholine (PC) in the embryos of *Brassica napus* seeds (Lu et al., 2018). Lipidomic has also been used to evaluate the characteristics of FAs and lipid species in fish. For example, 622, 530, and 513 lipids were identified in the muscle, head, and viscera of tilapia, respectively (He et al., 2021). It is also used to unravel the polar lipid and fatty acid composition in some algae, such as *Chondrus crispus* (Melo et al., 2015) and *Chlamydomonas nivalis* (Lu et al., 2012). However, there is a lack of research on the comparative lipidomics in Thraustochytrids treated with cold stress, particularly in FAs high-yield strains of *Aurantiochytrium* sp.

In this study, we hypothesized that cold stress (15°C, 5°C) accompanied with staged temperature control could enhance the yield of PUFAs. More specifically, cold stress would cause significant changes in fatty acids profiles in *Aurantiochytrium* sp., and some responsive genes might regulate these changes. To test our hypotheses, we performed an integrated transcriptomic and lipidomic analysis of the response of *Aurantiochytrium* sp. to cold stress and compared the physiological responses (FA synthesis, lipid metabolism, and regulatory processes). Two-staged temperature control in the *Aurantiochytrium* sp. fermentation for producing PUFAs was used. The results of this research propose a potential strategy to construct the DHA-high yield genetic engineered strain, thus creating a new proxy to improve the production yield and efficiency.

## Materials and methods

### Cultures and cultural conditions

*Aurantiochytrium* sp. SZU445, previously obtained by mutagenesis of *Aurantiochytrium* sp. under UV radiation, was used in this study (Liu et al., 2020). The strain culture medium and conditions were similar to those used in our prior study [natural seawater (salinity: 30.75∼33.19%), 1.50 g/L peptone, 20.00 g/L glucose, 0.025 g/L KH_2_PO_4_, 1.00 g/L yeast extract, and cultivated in 500 mL flask with 300 mL medium] (Liu et al., 2020). To analyze the response of *Aurantiochytrium* sp. SZU445 to low temperature, the strain was incubated at 25°C and 200 rpm for 84 h in a conical flask (stable growth phase), and some conical flasks were removed at 5 and 15°C from 84 to 144 h for FA composition, lipidomics, and transcriptomics analysis. Three treatments at 25, 15, and 5°C for the samples named Th_25, Th_15, and Th_5, respectively, were carried out.

### Dry cell weight and fatty acids analysis

Three parallel cell samples were collected from each temperature at regular intervals to analyze the biomass and growth curves. Cells were collected using a centrifuge at 4°C and 21,300 rcf for 5 min; next, cell pellets were lyophilized in a freeze dryer to calculate Dry cell weight (DCW), and freeze-dried biomass was harvested and stored for use. The freeze-dried biomass was ground into a fine powder for total lipid extraction based on the Bligh–Dyer method (Bligh and Dyer, 1959) and analyzed by an Agilent 7890-5975 gas chromatography-mass spectrometer (GC-MS) with H_2_ as the carrier gas using an Agilent J&W HP-5MS column (30 m × 250 μm i.d., 0.25 μm film thickness; Agilent Technologies, Palo Alto, United States) (Hartig, 2008). Inlet and detector temperatures were set to 250 and 260°C, respectively. The temperature program was as follows: initial temperature 60°C for 5 min, increased at 25°C/min to 180°C, increased to 240°C at a rate of 3°C/min and held for 1 min, and increased at 5°C/min to 250°C. In addition, the full scan mode was used for GC-MS detection.

### Sample pre-treatment and liquid chromatography electrospray ionisation tandem mass spectrometry-based lipidomics analysis

One sample (20 mg) was homogenized with a 1 mL mixture (including methanol, MTBE, and internal standard mixture) and a steel ball. The steel balls and whirls were removed from the mixture for 15 min. Next, 200 μL of water was added, and the mixture was whirled for 1 min, followed by centrifugation at 21,300 rcf and 4°C for 10 min. The supernatant (300 μL) was then extracted and concentrated. The powder was dissolved in 200 μL mobile phase B and stored at −80°C. Finally, the dissolving solution was placed in a sample bottle for LC-MS/MS analysis.

The sample extracts were analyzed using an LC-ESI-MS/MS system (UPLC, ExionLC AD^1^ ; MS, QTRAP^®^ System^2^). The analytical conditions were as follows, UPLC: column, Thermo Accucore™ C30 (2.6 μm, 2.1 mm × 100 mm); solvent system, A: acetonitrile/water (60/40, V/V, 0.1% formic acid, 10 mmol/L ammonium formate), B: acetonitrile/isopropanol (10/90 V V/V, 0.1% formic acid, 10 mmol/L ammonium formate); gradient program, A/B (80:20, V/V) at 0 min, 70:30 V/V at 2.0 min, 40:60 V/V at 4 min, 15:85 V/V at 9 min, 10:90 V/V at 14 min, 5:95 V/V at 15.5 min, 5:95 V/V at 17.3 min, 80:20 V/V at 17.3 min, 80:20 V/V at 20 min; flow rate, 0.35 mL/min; temperature, 45°C; Injection volume: 2 μL. The effluent was alternatively connected to an ESI-triple quadrupole linear ion trap (QTRAP)-MS.

LIT and triple quadrupole (QQQ) scans were acquired on a triple QTRAP-MS, QTRAP^®^ LC-MS/MS System, equipped with an ESI Turbo Ion-Spray interface, operating in positive and negative ion mode and controlled by Analyst 1.6.3 software (Sciex). The ESI source operation parameters were as follows: ion source, turbo spray; source temperature, 500°C; ion spray voltage (IS), 5,500 V (positive), −4,500 V (negative); ion source gas 1 (GS1), gas 2 (GS2), and curtain gas (CUR) were set at 45, 55, and 35 psi, respectively; and the collision gas (CAD) was medium. Instrument tuning and mass calibration were performed with 10 and 100 μmol/L polypropylene glycol solutions in the QQQ and LIT modes, respectively. QQQ scans were acquired as MRM experiments with a collision gas (nitrogen) set at 5 psi. DP and CE for individual MRM transitions were performed with further optimization of DP and CE. A specific set of MRM transitions was monitored for each period, according to the metabolites eluted within this period.

### Lipid identification

For lipid identification, the MS/MS spectrum of each feature was searched using MS-DIAL software with an integrated LipidBlast database (including HMDB, PubChem CID, CAS, ChEBI, and Metlin). In addition, lipid species were distinguished by using strict criteria: high mass accuracy of precursor ion (<0.01 Da), fragment ion (<0.05 Da), and ion intensity score involved in the consistency of the MS/MS fragmentation. The parameters RT tolerance, MS1 (primary MS profile) accurate mass tolerance, MS2 (secondary MS profile) accurate mass tolerance, and identification score cutoff value were set at 5 min, 0.01, 0.05, and 80.00%, respectively. Screening of differentially expressed lipids fulfilled the criterion of a cutoff value of log_2_ fold change (relative intensity) >1.5 and <-1.5 (*P* < 0.05).

### RNA extraction, transcriptomic analysis, and qRT-qPCR analysis

Samples were collected at 144 h after incubation at 25, 15, and 5°C for RNA extraction. Total RNA extraction and RNA quality was tasted by a 2100 Bioanalyzer (Agilent, Palo Alto, United States) and quantified using an ND-2000 (NanoDrop Technologies, Wilmington, DE, United States) (Supplementary Table 4). Then sequencing of each sample were performed on the Illumina platform (PE150) at BGI, Shenzhen, China. The cDNA library was constructed following the instructions of the manufacturer using the TruSeq™ RNA Sample Prep Kit from Illumina (San Diego, CA, United States) using 1 μg of total RNA. mRNA was isolated according to the polyA selection method by oligo(dT) beads and fragmented by fragmentation buffer first. Second, double-stranded cDNA was synthesized using a SuperScript double-stranded cDNA synthesis kit (Invitrogen, Carlsbad, CA, United States) with random hexamer primers (Illumina). Then, the synthesized cDNA was subjected to end-pair, phosphorylation, and “A” base addition fragments of 200-300 bp on 20% Low Range Ultra Agarose followed by PCR amplification using Phusion DNA polymerase (NEB) for 15 PCR cycles. After quantification by TBS380, the paired-end RNA-seq sequencing library was sequenced with Illumina HiSeq X ten (2 × 150 bp read length). Clean reads were obtained by removing the reads containing adapter, poly-N, and low-quality reads from the raw data. At the same time, Q20, Q30, GC content, and the sequence duplication level of clean reads were calculated. High-quality clean reads were used for transcriptome profiling analysis. The raw paired-end reads were trimmed and quality controlled by SeqPrep^3^ and Sickle^4^ with default parameters. Then, clean reads were separately aligned to the reference genome with an orientation mode using HISAT^5^ software. The mapped reads of each sample were assembled by StringTie^6^ in a reference-based approach. When identifying differentially expressed genes (DEGs) between two different samples, the expression level of each transcript was calculated according to the transcripts per million reads (TPM) method. RSEM^7^ was used to quantify gene abundances. R statistical package software EdgeR (Empirical Analysis of Digital Gene Expression in R)^8^ was utilized for differential expression analysis. qRT-PCR analysis was performed on a CFX Connect real-time system using SYBR Green qPCR Master Mix and a Bio-Rad CFX Real-Time PCR system, and 18S rDNA (GME13964_g) was used as the internal standard (Supplementary Table 2). The primers used for qRT-PCR were designed using Primer 3 software (Supplementary Table 3). The 2^–ΔΔCT^ method was used to analyze relative gene expression (Xiong et al., 2015).

Gene Annotation, GO Enrichment, and Kyoto Encyclopedia of Genes and Genomes (KEGG) Analysis. Gene function was annotated based on several databases, including Nr (NCBI non-redundant protein sequences), Nt (NCBI non-redundant nucleotide sequences), Pfam (protein family), COG (clusters of orthologous protein of proteins), Swiss-Prot (a manually annotated and reviewed protein sequence database), KO (KEGG ortholog database), and GO (gene ontology). Functional enrichment analysis, including GO and KEGG, was performed to identify which DEGs were significantly enriched in GO terms and metabolic pathways at a Bonferroni-corrected *P*-value of ≤0.05 compared with the whole-transcriptome background. GO functional enrichment and KEGG pathway analysis were carried out by Goatools^9^ and KOBAS.^10^

### Statistical analysis

All experimental data are presented as mean ± standard deviation, representing at least three independent experiments. GraphPad Prism version 8 (GraphPad Software, San Diego, CA, United States) was used to perform the statistical visualizations and analyses, and Adobe Illustrator CS (Adobe, San Jose, CA, United States) was used to map the metabolic network of fatty acids. Differences between mean values were analyzed by one-way analysis of variance using Tukey’s multiple range *post-hoc* test in GraphPad. Differences were considered statistically significant at *P < 0.05, ^**^P < 0.01, and ^***^P < 0.001.

## Results and discussion

### Effects of low temperature on fatty acid composition of *Aurantiochytrium* sp.

Temperature substantially affected the growth of *Aurantiochytrium* sp. SZU445 (shown as Supplementary Figures 1A,B). Notably, the final yield (144 h) of biomass with the 15°C low-temperature treatment (3.96 mg/mL) was 1.20 times and 1.13 times more than those of the 5°C low-temperature and 25°C normal-temperature treatments, respectively. The possible explanation could be that low temperature induced the cell enlargement and thus stimulated the intracellular calcification (Sorrosa et al., 2005). For the FAs production, the total yields of 1.04 mg/mL [59.63% total fatty acids (TFAs)], 1.25 mg/mL (52.79% TFAs), and 0.87 mg/mL (49.41% TFAs) PUFAs were accumulated at 5, 15, and 25°C, respectively. These results indicated that 15°C was more conducive to the accumulation of PUFAs yield. In addition, FA composition profiles of *Aurantiochytrium* sp. SZU445 cells incubated at different temperatures were also analyzed (Supplementary Figure 1C). The TFAs extracted from the 15°C low-temperature treatment was the highest, accounting for 59.50% of DCW, which is 1.18 times higher than in the 25°C treatment (50.30% DCW), and 1.12 times higher than in the 5°C treatment (53.10% of DCW). Moreover, a significant increase was observed in the incubation temperature of 5°C for the accumulation of DHA (C22:6) (50.43% of TFAs), palmitic acid (PLA) (C16:0) (27.54% of TFAs), and DPA (C22:5) (5.10% of TFAs). The contents of PLA with the 5°C low-temperature treatment was enhanced 1.42 times and 1.29 times compared with that of the 15 and 25°C treatment. PLA is the most commonly observed SFA and serves as a precursor for longer FAs. In addition, palmitoylation is crucial for the membrane localization of many proteins. This result was different from that of Ma et al. (2015) reported that the PLA content decreased from 40.53 to 33.38%, but it was similar to the increase from 51.05 to 53.53% in *Schizochytrium* sp. TIO01 under low-temperature treatments (Hu et al., 2020). In this study, the increase in PLA accumulation may contribute to a longer chain FA synthesis under low-temperature treatment. For DHA accumulation, the content with the 5°C low-temperature treatment increased 1.16 times and 1.25 times compared with that of the 15°C low-temperature treatment and the 25°C normal-temperature treatment, respectively. It was different from *Thraustochytriidae* sp. Z105 (15–25°C) (Zhou et al., 2010) and *Schizochytrium* sp. TIO01 (16°C) (Hu et al., 2020). Many studies have reported that low temperatures can enhance lipid production and the proportion of unsaturated fatty acids, but limit cell growth (Supplementary Figure 1D) (Gounot, 1991; Taoka et al., 2009). In our study, the TFAs yield and biomass were promoted with 15°C treatment, while for the specific FA species such as PLA, DPA, and DHA, were accumulated more under 5°C treatment.

### Lipidomic profiling of *Aurantiochytrium* sp. under cold stress

**FIGURE 1:**
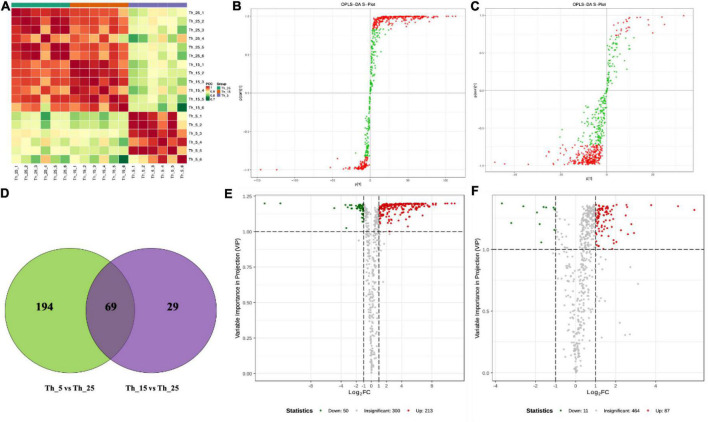
Lipidomic analysis of *Aurantiochytrium* sp. SZU445 under low temperature. **(A)** Correlation between samples, **(B)** OPLS-DA S-plot analysis of Th_5 vs. Th_25, **(C)** OPLS-DA S-plot analysis of Th_15 vs. Th_25, **(D)** venn diagram of different metabolites in each group, **(E)** differential metabolites volcano map in Th_5 vs. Th_25, **(F)** differential metabolites volcano map in Th_15 vs. Th_25 (The samples treated with 25, 15, and 5°C were named Th_25, Th_15, and Th_5, respectively).

To further determine and evaluate the changes in the overall lipid composition in response to various low-temperature treatments, lipidomic profiling of *Aurantiochytrium* sp. was performed using LC-ESI-MS/MS. A total of 563 lipids were identified, including FAs (61), glycerolipids (GLs) (281), glycerophospholipids (GPs) (181), and sphingolipids (SLs) (39) (Figure 1A).

#### Differential metabolites analysis

Orthogonality partial least squares-discriminant analysis (OPLS-DA) was used to detect differential metabolites (DEMs). The DEMs in the Th_5 vs. Th_25 comparison group (*R*^2^X = 0.735, *R*^2^Y = 1) were more significant than those in Th_15 vs. Th_25 group (*R*^2^X = 0.66, *R*^2^Y = 0.99) (Figures 1B,C). There were 263 DEMs in the Th_5 vs. Th_25 comparison group with 213 upregulated metabolites and 50 downregulated metabolites (Figure 1D). However, in Th_15 vs. Th_25, there were 98 DEMs with 87 upregulated metabolites and 11 downregulated metabolites (Figures 1E,F). Comparing DEMs among the Th_5 vs. Th_25 and Th_15 vs. Th_25 groups, 69 metabolites were identical, and 194 metabolites were only detected in the Th_5 vs. Th_25 group, and 29 metabolites were only detected in the Th_15 vs. Th_25 comparison group (Figure 1D). Most notable was that the number of DEMs in the Th_5 vs. Th_25 comparison group was much higher than that in the Th_15 vs. Th_25 group.

The DEMs identified in Th_5 vs. Th_25 comparison group include 144 species of GP (138 upregulated and 6 downregulated), 66 species of GL (38 upregulated and 28 downregulated), 37 species of FAL (fatty acyl) (34 upregulated and 3 downregulated), and 20 species of SL (8 upregulated and 12 downregulated) (Supplementary Figure 2A). The top 20 KEGG enrichment analyses of these DEMs showed that the phospholipase D signaling pathway, phosphatidylinositol (PI) signaling system, and GP metabolism were the main responses to low-temperature stress in lipid metabolism (Supplementary Figure 2C), which may be because GP composition affects cold tolerance ability (Tomcala et al., 2006). In the Th_15 vs. Th_25 comparison group, there were 53 species of GP (44 upregulated and 9 downregulated), 18 species of GL (upregulated), 14 species of FAL (13 upregulated and 1 downregulated), and 9 species of SL (upregulated) identified (Supplementary Figure 2B). Additionally, the sphingolipid metabolism, phospholipase D signaling pathway and GP metabolism in KEGG enrichment were the main responses to low temperature in lipid metabolism (Supplementary Figure 2D), which was lower than the Th_5 vs. Th_25 group in metabolite numbers and different significance. This indicates that an increase in the relative proportions of lipid classes in the lipid bilayer and the degree of unsaturation in glycerophospholipids to increase membrane fluidity modulation are important strategies for cell adaptation to low-temperature stress (Harwood, 1991; Tomcala et al., 2006).

#### Variation tendency of differential metabolites in *Aurantiochytrium* sp. under cold stress

Six classes with 350 DEMs were obtained by comparing the metabolites at different incubation temperatures based on K-means clustering algorithm analysis (Supplementary Figure 3). The content of candidate metabolites in classes 1, 2, and 5 presented an increasing trend at 5°C. By contrast, 16 DEMs in class 4 showed a decreasing trend, such as cholesterol lipids (16:0). These results indicated that 5°C treatment increased the types of long-chain lipids in *Aurantiochytrium* sp. SZU 445, while reducing the types of short-chain saturated pantothenic acid, regulates the degree of lipids that are unsaturated to maintain the normal membrane lipid physical state to adapt to low temperatures (Zhu et al., 2007). In classes 3 and 6, incubation at 15°C presented an increasing trend for the categories of metabolites, such as phosphatidylethanolamine (PE) (18:1_18:2) and triglycerides (16:0_18:2_18:3), while a decreasing trend at 5°C was observed. These results indicate that incubation at 15°C was more conducive to an increase in the types of medium-chain lipids.

#### Comparative analysis of lipids under cold stress

**FIGURE 2:**
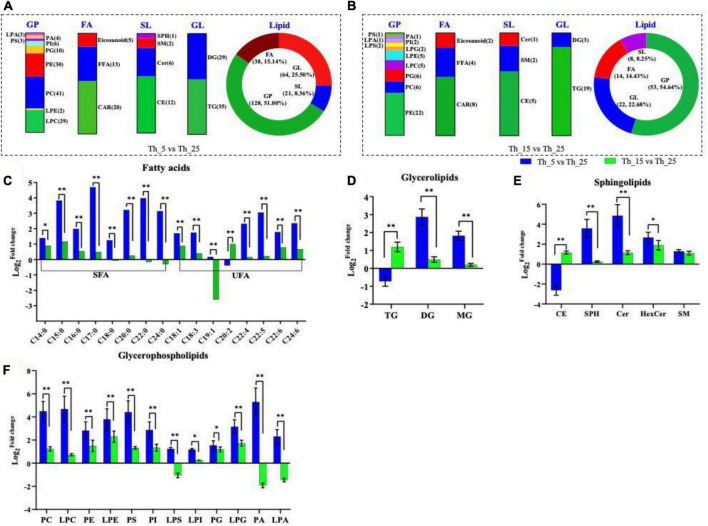
Lipid composition under different temperature treatment. Differential identified lipid numbers in Th_5 vs. Th_25 **(A)** and Th_15 vs. Th_25 **(B)**. Comparison of different fatty acids **(C)**, glycerolipids **(D)**, sphingolipids **(E)**, glycerophospholipids **(F)** among h_5 vs. Th_25 (blue column) and Th_15 vs. Th_25 (green column). FAL, fatty acyls; GL, glycerolipids; SL, sphingolipids; GP, glycerophospholipids; TG, triglycerides; DG, diglycerides; MG, monoglyceride; CE, cholesterol; SPH, sphingosine; Cer, ceramide; HexCer, glycoceramides; SM, sphingomyelin (The samples treated with 25, 15, and 5°C were named Th_25, Th_15, and Th_5, respectively). (*P < 0.05; **P < 0.01).

In the Th_5 vs. Th_25 comparison group, there were 251 species lipids, including fatty acyls (FAL) (38, 15.14%), glycerolipids (GL) (64, 25.50%), sphingolipids (SL) (21, 8.36%), and glycerophospholipids (GP) (128, 51.00%) changed significantly (Figure 2A). While, in the Th_15 vs. Th_25 comparison group, there were only 97 species lipids, including FAL (14, 14.43%), GL (22, 22.68%), SL (8, 8.25%), and GP (53, 54.64%), changed significantly (Figure 2B).

##### Increase in fatty acyl accumulation response to cold stress

Fatty acyl composition in the membrane is one of the critical factors determining low-temperature sensitivity (Larkindale and Huang, 2004). In *Aurantiochytrium* sp. SZU 445, composition and content (% TFAs) of FAL also changed in response to low temperatures. In the Th_5 vs. Th_25 comparison group, almost all SFAs [myristic acid (C14:0), pentadecanoic acid (C15:0), PLA (C16:0), heptadecanoic acid (C17:0), stearic acid (C18:0), arachidic acid (C20:0), behenic acid (C22:0), and tetradecanoic acid (C24:0)] were upregulated (Figure 2C).

Unsaturated fatty acids (UFAs), [oleic acid (C18:1), linolenic acid (C18:3), docosatetraenoic acid (C22:4), DPA (C22:5), tetradecahexaenoic acid (C24:6), and DHA (C22:6)] were also upregulated under low-temperature. The content of DPA and DHA were upregulated 8.31-fold and 3.42-fold in the Th_5 vs. Th_25 group, and a 1.17-fold change and 1.73-fold change were observed in the Th_15 vs. Th_25 group. These changes were higher than those of the *Schizochytrium* sp. TIO01 at 16°C, whose PLA increased from 51.05 to 53.53% and DHA increased from 43.00 to 65.00% (1.51-fold) (Hu et al., 2020), and higher than those of *Aurantiochytrium* sp. SD116, and DPA increased from 8.18 to 9.27% at 15°C, and DHA increased from 43.10 to 52.48% (1.22-fold) (Ma et al., 2015). There are few reports on lipid changes under the 5°C-lower temperature treatment. Only in *Oblongichytrium* RT2316-13 were the genes related to EPA and DHA synthesis upregulated at 5°C (Paredes et al., 2020), which is the local lipid metabolism profile. In addition, eicosadienoic acid (EA) (20:2) was significantly higher than that in the Th_15 vs. Th_25 comparison group (2.00-fold). At 5°C, SFA and UFA were both upregulated, the possible explanation could be that more EA was used to synthesize docosatetraenoic acid and DPA (Renard-Merlier et al., 2009). Above all, low temperature greatly affected the FA composition, especially the content of PUFA.

##### Increase in glycerophospholipids accumulation response to cold stress

Glycerides (GLs), including triglycerides (TG), diglycerides (DG), and monoglyceride (MG), were significantly upregulated under 5°C treatment in *Aurantiochytrium* sp. SZU445 (Figure 2D). And in the Th_5 vs. Th_25 group, the changes in DG and MG were 5.79 and 9.10 times higher than those in the Th_15 vs. Th_25 group. These results are consistent with the desaturation of GLs being an especially effective strategy against low temperatures, which plays an important role in the membrane structure and dynamics (Varkonyi et al., 2002). Hence, an increase in DG proportion may contribute to *Aurantiochytrium* sp. membrane fluidity.

Sphingolipids (SLs), including cholesterol (CE), sphingosine (SPH), ceramide (Cer), glycoceramides (HexCer), and sphingomyelin (SM) were upregulated in the two groups, except for CE in the Th_5 vs. Th_25 group (Figure 2E). The bulky, rigid tetracyclic structure of CE makes the cell membrane less fluidic and less permeable to ions and solutes (Haines, 2001; Chung et al., 2015). Therefore, decreased CE accumulation may help change the membrane structure and dynamics in response to cold stress. Additionally, glycerophospholipid (GPs), including pyruvate carboxylase (PC), lysophosphatidylcholine (LPC), PE, lysophosphatidylethanolamine (LPE), phosphatidylserine (PS), lysophosphatidylserine (LPS), PI, lysophosphatidylinositol (LPI), phosphatidylglycerol (PG), lysophosphatidylglycerol (LPG), phosphatidic acid (PA), and LPA, constitute the core metabolome responsive to the stresses (Sivankalyani et al., 2016; Pan et al., 2018), and showed significant upregulation in the Th_5 vs. Th_25 group and higher than that in the Th_15 vs. Th_25 group, such as PC, PS, and PA (Figure 2F). PC, as a substrate for membrane-bound desaturases in the endoplasmic reticulum, makes important contributions to the formation of a pool of free PUFAs (Browse and Somerville, 1991). PC is also a precursor for the synthesis of glycerolipids in plastid membranes (Ohlrogge and Browse, 1995). Hence, the upregulation of PC in *Aurantiochytrium* sp. SZU 445 shows that the amount of polyunsaturated PC is correlated with cold tolerance (Kinney et al., 1987). In addition, PC serves as a precursor for the synthesis of complex lipids (Testerink and Munnik, 2005) and is transiently and rapidly synthesized in response to stress in *Aurantiochytrium* sp. SZU 445. The results indicate that incubation at 5°C stimulated the upregulation of GL, SL, and GP content to respond cold stress.

### Transcriptome profiling of *Aurantiochytrium* sp. under cold stress

The Illumina RNA-seq approach was used to detect the relationships between lipid metabolism and the relevant metabolic genes, to determine the mechanism of the *Aurantiochytrium* sp.’s response to cold stress. Among all transcripts, 13,734 genes were identified based on the *Aurantiochytrium* sp. SZU445 genome (Zhu et al., 2020). A total of 11,464 DEGs (*P*-adjust < 0.05 and | log2FC| ≥ 1) were identified. Among the DEGs, 4658 and 2555 genes were upregulated in the Th_5 vs. Th_25 and Th_15 vs. Th_25 groups, respectively. In addition, 4,829 and 3,090 genes were downregulated in response to low temperatures (Supplementary Figures 4A,D).

#### Transcriptomics analysis revealed the perception and transduction of cold stress signals

Interestingly, there were 247 DEGs enriched in the lipid metabolism pathway in the Th_5 vs. Th_25 group, which had 71 DEGs more than that in Th_15 vs. Th_25 group (176) (Supplementary Figures 4C,F). This indicated a complementary, positive agreement between transcriptomic and lipidomic analyses.

In *Aurantiochytrium* sp. SZU445, the perception and transduction of low-temperature signals is an immediate acclimation to cold stress. Extracellular signal-regulated kinase (ERK) which regulates cellular activities in the signal transduction was identified with 1.66-fold and 1.25-fold upregulation in the Th_5 vs. Th_25 and Th_15 vs. Th_25 groups, respectively, which indicated the role of ERK in the cold stress response. The histidine kinase was upregulated 1.37-fold in the Th_5 vs. Th_25 group and downregulated 0.61-fold in the Th_15 vs. Th_25 group, which was involved in the transduction and perception of cold stress signals by regulating the RNA helicase genes and lipid desaturase (Suzuki et al., 2000). The cAMP-PKA pathway also plays a major role in the response to cold stress (Liu et al., 2017). Tyrosine kinase, a key enzyme in the cAMP-PKA pathway, was found to be upregulated by 1.44-fold in the Th_5 vs. Th_25 group. Cold stress also induces cellular ion homeostasis. For example, activation of ion channels due to cold may result from physical alterations in the cell structure (Xiong et al., 2002). Calcium/calmodulin-dependent protein kinase can regulate the activation of ion channels (Wang et al., 1998) and was confirmed and upregulated 2.85-fold and 1.34-fold in the Th_5 vs. Th_25 and Th_15 vs. Th_25 groups, respectively. Through the upregulation of ERK and the cAMP-PKA pathway, the accumulation of lipids and FAs was also enriched under low temperatures.

#### Transcriptomics profile of genes involved in lipid metabolism

In the two groups, the most enriched pathways included FA metabolism (74–55 DEGs), FA degradation (72–53 DEGs), glycerophospholipid metabolism (46–39 DEGs), glycerolipid metabolism (40–26 DEGs), sphingolipid metabolism (37–19 DEGs), biosynthesis of antibiotics (88–64 DEGs), FA biosynthesis (30–25 DEGs), and elongation (27–18 DEGs) (Supplementary Figures 4B,C,E,F). This result indicated that under the 5°C incubation, more genes and pathways were induced in response to cold stress. The detailed mechanisms are described as follows.

**FIGURE 3:**
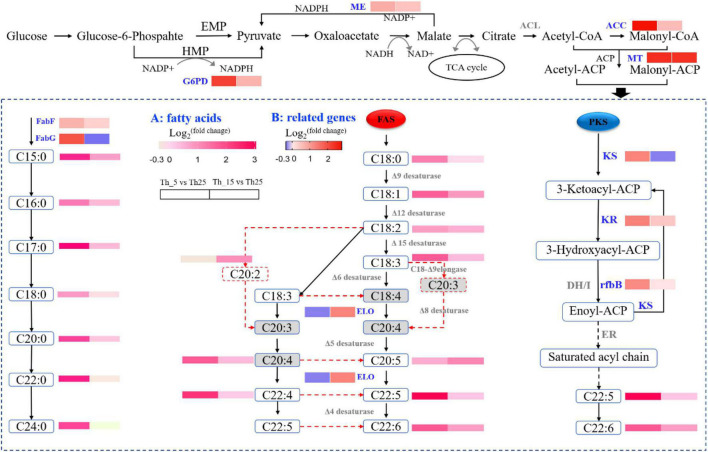
Diagrammatic representation of significantly differentially expressed genes related to fatty acids biosynthesis pathway under 5 and 15°C treatment (A: the change of key metabolites; B: the change of key genes in the pathway). ME, malic enzyme; ACC, acetyl-CoA carboxylase; MT, malonyl-CoA:ACP transacylase; G6PD, glucose-6-phosphate 1-dehydrogenase; KS, 3-ketoacyl-CoA synthase; KR, ketoreductase; rfbB, dTDP-glucose 4,6-Dehydratase; ELO, fatty acid elongase 3; FabF, 3-oxoacyl-[acyl-carrier-protein] synthase II; FabG, 3-oxoacyl-[acyl-carrier protein] reductase; A, fatty acids; B, related genes.

For FA metabolism upon treatment at low temperature, SFAs and UFAs showed an increased level, specifically at 5°C incubation (Figure 3). Some key genes were upregulated for key enzyme synthesis under low-temperature stress (Table 1). Such as Acetyl-CoA and NADPH, as precursors for the biosynthesis of SFAs and UFAs, are necessary in a continuous manner for central carbon metabolism (Napier, 2002). As the key enzyme, ATP citrate lyase (ACL) was not identified in our study, and pyruvate dehydrogenase (PDH) was upregulated 2.28-fold and 1.57-fold in the Th_5 vs. Th_25 and Th_15 vs. Th_25 groups, respectively (Table 1). In the NADPH generation, malic enzyme (ME) and glucose-6-phosphate 1-dehydrogenase (G6PD) play important roles in FA accumulation involved in the pentose phosphate pathway to produce NAPDH (Liang and Jiang, 2015), and were upregulated 1.69-fold and 4.50-fold in the Th_5 vs. Th_25 group, which were higher than those in Th_15 vs. Th_25 group (1.38-fold and 1.82). In addition, acetyl-CoA carboxylase (ACC) and malonyl-CoA:ACP transacylase (MT), converting acetyl-CoA to acetyl-ACP and malonyl-ACP, were also upregulated 6.87-fold and 2.41-fold in the Th_5 vs. Th_25 group, respectively, which were higher than in the Th_15 vs. Th_25 group (1.49-fold and 5.21 fold, respectively). Most notable was at low temperatures, the genes related to acetyl-CoA and NADPH for FA synthesis were upregulated, especially at 5°C.

**TABLE 1:** Transcriptomics of the expression of genes related to fatty acids synthesis and metabolism.

Gene ID	Name	Description	Th_5 vs. Th_25 (Log_2_ fold change)	Th_15 vs. Th_25 (Log_2_ fold change)
GME12288_g	ACC	Acetyl-CoA carboxylase	2.78	0.58
GME6333_g	FAS	Fatty acid synthase	−1.30	0.63
GME4772_g	ELO3	Fatty acid elongase 3	−0.31	1.29
GME4457_g	VLCR	Very-long-chain 3-oxoacyl-CoA reductase	1.20	–
GME5355_g	ME2	Malate dehydrogenase (oxaloacetate-decarboxylating)	0.76	0.46
GME4772_g	KS	3-ketoacyl-CoA synthase	1.29	−0.31
GME1766_g	rfbB	dTDP-glucose 4,6-Dehydratase	1.21	0.04
GME12428_g	KR	Ketoreductase	1.32	0.43
GME2060_g	ACADM	Acyl-CoA dehydrogenase	1.97	–
GME227_g	ECH	Enoyl-CoA hydratase	3.29	1.32
GME4106_g	G6PD	Glucose-6-phosphate 1-dehydrogenase	2.17	0.86
GME6118_g	HK	Glycolysis hexokinase	1.84	1.56
GME12318_g	PFKA	6-phosphofructokinase	−3.44	0.33
GME11484_g	PGAM	Phosphoglycerate mutase	−1.37	0.55
GME9101_g	PGD	6-phosphogluconate dehydrogenase	1.60	0.94
GME4004_g	PDH	Pyruvate dehydrogenase	1.19	0.65
GME2592_g	PC	Pyruvate carboxylase	−3.33	–
GME13163_g	LSC1	Succinyl-CoA synthetase	0.63	–
GME5879_g	ACS	Acetyl-CoA synthetase	−1.07	−0.98
GME502_g	FabD	Malonyl coenzyme A-acyl carrier protein transacylase	2.42	2.38
GME11352_g	Mat	Malonyl-CoA/methylmalonyl-CoA synthetase	0.88	0.71
GME3512_g	ACSL	Long-chain acyl-CoA synthetase	1.73	0.67
GME10740_g	ACAA1	Acetyl-CoA acyltransferase	0.32	−0.69
GME8911_g	ACOX	Acyl-CoA oxidase	0.29	–
GME11557_g	EHHADH	3-hydroxyacyl-CoA dehydrogenase	0.60	−0.58
GME11731_g	ECI1	Delta3-Delta2-enoyl-CoA isomerase	0.57	–
GME9935_g	FabF	3-oxoacyl-[acyl-carrier-protein] synthase II	−1.76	0.30
GME9912_g	FabG	3-oxoacyl-[acyl-carrier protein] reductase	1.93	–
GME10958_g	HACD	Very-long-chain (3R)-3-hydroxyacyl-CoA dehydratase	−0.55	0.37
GME11973_g	TECR	*Trans*-2-enoyl-CoA reductase	0.28	0.31

The contents of SFA and PUFA were increased under 5°C in *Aurantiochytrium* sp. SZU445 (Figure 3). In addition, transcriptomic and qRT-PCR analyses indicated that genes involved in the SFA synthesis pathway, fatty acid synthase (FAS), and polyketide synthase (PKS) pathways were upregulated (Figure 3; Table 1). In the PKS pathway, the key enzyme genes 3-ketoacyl-CoA synthase (KS) and ketoreductase (KR) were identified, with 2.44-fold and 2.50-fold upregulation in the Th_5 vs. Th_25 group, respectively, which were higher than in the Th_15 vs. Th_25 group (0.81- fold and 1.35-fold, respectively). Nevertheless, dehydrase/isomerase (DH/I) and enoyl reductase (ER) were not identified. Additionally, another similar DH, dTDP-glucose 4,6-Dehydratase (rfbB) was identified in our prior study (Zhu et al., 2020), was also identified in this study, with 2.31 and 1.03-fold upregulation in the Th_5 vs. Th_25 and Th_15 vs. Th_25 groups, respectively. In the FAS pathway, FAS and fatty acid elongase 3 (ELO) were downregulated 0.41-fold and 0.81-fold, respectively at 5°C. However, FAS and ELO were upregulated by 1.55-fold and 2.45-fold, respectively at 15°C. Therefore, we hypothesize that the accumulation of DHA and other PUFAs was due to the overexpression of PKS pathway genes at 5°C; at 15°C, DHA and other PUFAs’ accumulation were upregulated through the overexpression of FAS pathway genes. This result was similar to that reported by Hu et al. (2020) for *Schizochytrium* sp. TIO01 at 15°C.

Unlike other studies, SFA accumulation increased significantly in *Aurantiochytrium* sp. SZU445 cells after incubation at 5°C (Figure 3). In the SFA synthesis pathway, 3-oxoacyl-[acyl-carrier-protein] synthase II (FabF) and 3-oxoacyl-[acyl-carrier protein] reductase (FabG) were upregulated 1.60-fold and 3.81-fold at 5°C, respectively, which were higher than those observed at 15°C. In addition, acyl-CoA dehydrogenase (ACADM) catalyzes the first step of FA β-oxidation degradation in peroxisomes and is overexpressed 3.92-fold at 5°C (Table 1). The third step of FA synthesis in mitochondria was also upregulated through enoyl-CoA hydratase (ECH) overexpression by 9.78-fold at 5°C. These results suggest that FA metabolism plays an important role in SFA synthesis and increases the synthesis of SFAs at 5°C.

##### Glycerophospholipid metabolism

**FIGURE 4:**
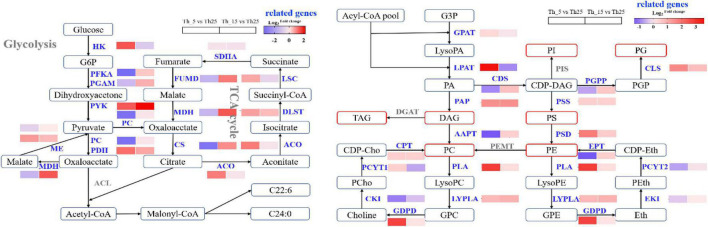
Changes in transcript abundance of genes involved in glycolysis, TCA cycle, and glycerophospholipid biosynthesis under 5 and 15°C treatment in *Aurantiochytrium* sp. SZU445. GPAT, lycerol-3-phosphate acyltransferase; LPAT, lysophospholipid acyltransferase; PAP, phosphatidic acid phosphatase; AAPT, aminoalcoholphosphotransferase; PEMT, phosphatidylethanolamine/phosphatidyl-N-methylethanolamine N-methyltransferase; DGAT, diacylglycerol O-acyltransferase; PSS, phosphatidylserine synthase; PSD, phosphatidylserine decarboxylase; PGP, phosphatidylglycerophospate; CLS, cardiolipin synthase; PLA, phospholipase A; LYPLA, lysophospholipase; GDPD, glycerophosphoryl diester phosphodiesterase; EKI, ethanolamine kinase; CKI, choline kinase; HK, hexokinase; PFKA, 6-phosphofructokinase; PGAM, phosphoglycerate mutase; SDHA, succinate dehydrogenase; FUMD, fumarate hydratase; MDH, malate dehydrogenase; CS, citrate synthase; ACO, aconitate hydratase; DLST, dihydrolipoamide succinyltransferase; LSC, succinyl-CoA synthetase. The columns in the figure represent the fold changes of gene expression in Th_5 vs. Th_25 and Th_15 vs. Th_25.

From G3P to synthesis PC, the enzyme genes, including glycerol-3-phosphate acyltransferase (GPAT), lysophospholipid acyltransferase (LPAT), and phosphatidic acid phosphatase (PAP), were upregulated by 1.22-fold, 11.96-fold, and 2.77-fold, respectively, at 5°C, which increased the accumulation of PA and DAG (Figure 4). However, aminoalcoholphosphotransferase (AAPT) was downregulated by 0.39-fold after incubation at 5°C, which was lower than that after incubation at 15°C (1.34-fold). Therefore, we speculated that PC was synthesized from PE, and phosphatidylethanolamine/phosphatidyl-N-methylethanolamine N-methyltransferase (PEMT) was overexpressed for PC accumulation. TAG was synthesized from DAG through diacylglycerol O-acyltransferase (DGAT), which was not identified in this study. Based on the decrease in content of TAG content, our assumption was that DGAT was downregulated in the TAG synthesis pathway. PE was synthesized from CDP-DAG through phosphatidylserine synthase (PSS), and phosphatidylserine decarboxylase (PSD). At 5°C, PSS and PSD increased 1.35-fold and 3.43-fold, respectively, which increased the accumulation of PE. PG can be synthesized from phosphatidylglycerophospate (PGP) through cardiolipin synthase (CLS), which was upregulated 3.01-fold at 5°C and 1.64-fold at 15°C. PIS upregulation was speculated by combining PI accumulation at low temperatures. In addition, during the synthesis of PE and PC, phospholipase A (PLA), lysophospholipase (LYPLA), and glycerophosphoryl diester phosphodiesterase (GDPD), which are involved in the first step of PC degradation, showed significant changes under low-temperature. At 5°C PLA, LYPLA, and GDPD showed an increase of 7.21-fold, 1.77-fold, and 6.68-fold, respectively. In the synthetic cycle of PE and PC, ethanolamine kinase (EKI) and choline kinase (CKI) involved in the first step of PE and PC synthesis, decreased (Figure 4), which indicated that the synthesis of PC and PE was mainly through PA and PS, rather than a synthetic cycle in response to cold stress. The essential components of the membrane, PC, PE, PA, PS, PI, and PG, were increased, which remodeled the lipid composition and adapted to cold stress (Zheng et al., 2016; Gu et al., 2017; Margutti et al., 2017).

#### Significant differentially expressed genes related to glycolysis and tricarboxylic acid cycle

Glycolysis can produce NADPH and acetyl-CoA for FA synthesis. When *Aurantiochytrium* sp. SZU445 was incubated at low temperatures, and genes involved in glycolysis, including hexokinase (HK), 6-phosphofructokinase (PFKA), and phosphoglycerate mutase (PGAM), were upregulated significantly (Figure 4). HK was upregulated by incubation at 5°C by 2.71-fold, which was higher than that at 15°C (0.53-fold), which was different from HK changes in *Penicillium* sp. and *Aspergillus glaucus* under cold stress (Kostadinova et al., 2011). This finding indicated that accumulating more G6P to produce NADPH for FA synthesis at 5°C (Liu et al., 2015). However, PFKA and PGAM were downregulated by 0.09-fold and 0.39-fold at 5°C, respectively, which were lower than that at 15°C (1.26-fold and 1.46-fold, respectively). This indicated that the glycolysis pathway was inhibited under the 5°C treatment and promoted under the 15°C treatment. These results differed from those of the glycolysis pathway downregulated under 16°C incubation (Hu et al., 2020). Therefore, in *Aurantiochytrium* sp. SZU445, glucose metabolism is mainly via the pentose phosphate pathway to produce more NADPH for FA synthesis at 5°C (Song et al., 2013).

In the TCA cycle, the first step was to convert pyruvate to oxaloacetate through PC, which was downregulated by 0.10-fold at 5°C and 0.89-fold at 15°C. Additionally, other key enzyme genes, including succinate dehydrogenase (SDHA), fumarate hydratase (FUMD), malate dehydrogenase (MDH), and citrate synthase (CS), were also downregulated by 0.83-fold, 0.35-fold, 0.37-fold, and 0.11-fold, respectively, at 5°C (Figure 4), indicating that the TCA cycle was inhibited to some extent under cold stress. However, aconitate hydratase (ACO), dihydrolipoamide succinyltransferase (DLST), and succinyl-CoA synthetase (LSC) were upregulated, which could increase succinate accumulation to produce the acetyl-CoA precursor citrate through the TCA cycle. Eventually, more acetyl-CoA was synthesized for FA synthesis (Sun et al., 2019), and TCA cycle inhibition was also consistent with our result in the first section that under 5°C treatment, cell growth was slow (Ma et al., 2017).

#### Verification of the gene expression through qRT-PCR

The RNA sequencing data demonstrated that the expression of FAs and other lipid genes was induced significantly by low temperature. For validating the gene expression level of the RNA-Seq data, 11 DEGs were selected for quantitative RT-PCR analysis. The results of gene expression levels obtained by qRT-PCR and RNA-Seq were highly consistent (*R*^2^ = 0.94) (Supplementary Figure 5).

### Multi-omics reveal the mechanism of *Aurantiochytrium* sp. SZU445 in response to cold stress

**FIGURE 5:**
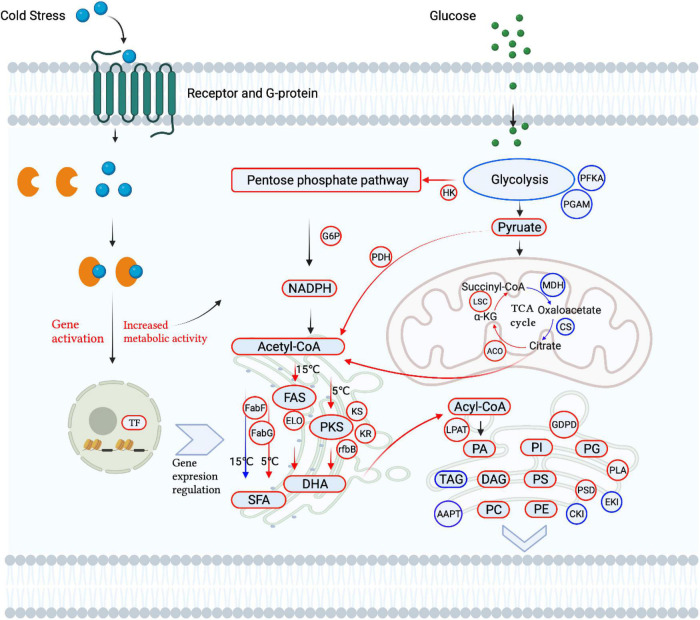
Schematic diagram of lipid metabolism affected by cold stress in *Aurantiochytrium* sp. SZU445.

The regulation network of lipid metabolism and gene expression involved in cold tolerance is shown in Figure 5, where red indicates upregulation and blue indicates downregulation. Under cold stress, glycolysis and the TCA cycle are downregulated, which is different from the regulation in plants (Dong et al., 2020). This indicates that cold stress reduces the cellular energy supply from glycolysis and the TCA cycle and inhibits the cell growth of *Aurantiochytrium* sp. SZU445. However, the pentose phosphate pathway was upregulated for NADPH and ATP for cell survival and FA synthesis, which was consistent with the cell growth performance and with what Ma et al. (2017) reported in *Aurantiochytrium* sp. under cold treatment. The accumulation of SFAs and PUFAs and some key genes were upregulated under cold stress. The FA synthesis pathway, FAS, and PKS were identified in *Aurantiochytrium* sp. SZU445, and the PKS pathway was the main pathway for synthesizing PUFAs at 5°C; the FAS pathway was the main pathway at 15°C. This finding was consistent with the results of Cheng et al. (2016) reported that *Aurantiochytrium* sp. CGMCC 6208 at 5°C. However, SFA synthesis was not downregulated. Almost all glycerophospholipids, including PA, PI, PG, PS, PC, PE, and DAG, were increased in remodeling membranes to respond to cold stress, for example, to improve the fluidity and integrity of the membrane (Gao et al., 2015; Barrero-Sicilia et al., 2017).

## Conclusion

In this study, active changes in lipids under cold stress were studied, and differential gene expression was explored in *Aurantiochytrium* sp. SZU445. The FA compositions between 5 and 15°C incubation were compared, and notably, at 15°C was suitable for the accumulation of PUFAs. Furthermore, upregulated gene expression of the FAS pathway (FAS and fatty acid elongase 3) and PKS pathway [3-ketoacyl-CoA synthase, ketoreductase and dTDP-glucose 4,6-Dehydratase (rfbB)] probably corresponded to 15 and 5°C treatment, respectively. 5°C treatment increased the types of long-chain lipids, and 15°C was more conducive to an increase in the types of medium-chain lipids. Additionally, glycolysis and the TCA cycle were inhibited under low temperature (5°C). Glycerophospholipid metabolism, including PA, PS, PG, PI, PC, and PE, was increased to enhance the fluidity and integrity of membranes to adapt to cold stress. This study innovatively identified genes for improving FA and lipid synthesis under cold stress, providing sufficient information for constructing high-yield strains based on genetic engineering.

## Data availability statement

The datasets presented in this study can be found in online repositories. The names of the repository/repositories and accession number(s) can be found below: NCBI BioSample–SAMN27608973, SAMN27608974, SAMN27608975, SAMN27608976, SAMN27608977, SAMN27608978, SAMN27608979, SAMN27608980, and SAMN27608981.

## Author contributions

YS: investigation, data curation, and review writing – original draft. ZH: conceptualization and review and editing. ZX, SL, TT, and WL: review and editing. XY: conceptualization and funding acquisition, review and editing, and supervision. All authors contributed to the article and approved the submitted version.

## Footnotes

1
https://sciex.com.cn/

2
https://sciex.com/

3
https://github.com/jstjohn/SeqPrep

4
https://github.com/najoshi/sickle

5
https://ccb.jhu.edu/software/hisat2/index.shtml

6
https://ccb.jhu.edu/software/stringtie/index.shtml?t=examole

7
http://deweylab.biostat.wisc.edu/rsem/

8
http://www.bioconductor.org/packages/2.12/bioc/html/edgeR.html

9
https://github.com/tanghaibao/Goatools

10
http://kobas.cbi.pku.edu.cn/home.do
