# Supplementary material for: Comparative transcriptomic and lipidomic analyses indicate that cold stress enhanced the production of the long C18–C22 polyunsaturated fatty acids in Aurantiochytrium sp
Source: Front Microbiol. 2022 Sep 20;13:915773. doi: 10.3389/fmicb.2022.915773 (PMC9530390; doi:10.3389/fmicb.2022.915773)
Supplement: Supplementary file 1 [file Table_1.docx]

**Table S1 The effect of temperature on bioactive compounds accumulation in microalgae**

| **Microalgae** | **Temperature** | **Fermentation strategy** | **Metabolite** | **Transcriptomic** | **Lipidomic** | **Reference** |
| --- | --- | --- | --- | --- | --- | --- |
| *Auxenochlorella protothecoides* UTEX2341 | 32 °C | One-step fermentation | Lipid | MYB3 and AP2-4 ↑ | / | (Yang et al., 2022) |
| *Auxenochlorella protothecoides* | 10 °C ,32 °C | One-step fermentation | FAs | KCS, KCR ↑ | / | (Xing et al., 2018) |
| *Chlorella* sp. L166 | 25 °C | One-step fermentation | ALA | pyruvate dehydrogenase ↑ | / | (Zheng et al., 2021) |
| *Chlorella* sp. UMACC 237 | 33 °C | One-step fermentation | FAs | Acetyl-CoA carboxylase ↓ | / | (Poong et al., 2018) |
| *Chlamydomonas reinhardtii* | 42 °C | Staged temperature control method | Lipid | DAG Gacyltransferase ↑ | DAG, TAG ↑ | (Legeret et al., 2016) |
| *Cylindrotheca closterium* | 11 °C | One-step fermentation | PUFAs | / | / | (Almeyda et al., 2020) |
| *Isochrysis galbana* | 35 °C | One-step fermentation | TAG | dihydrolipoamide succinyltransferase ↑ | TAG ↑ | (Cao et al., 2020) |
| *Isochrysis galbana* | 17 °C | One-step fermentation | DHA | / | / | (Aussant et al., 2018) |
| *Nannochloropsis salina* | 5 °C | Staged temperature control method | EPA | / | TAG ↑ | (Gill et al., 2018) |
| *Picochlorum* sp. (BPE23) | 40 °C | One-step fermentation | / | / | / | (Barten et al., 2022) |
| *Scenedesmus* sp. NREL 46B-D3 | 13 °C | One-step fermentation | FAs | KAS ↑ | TAG ↑ | (Calhoun et al., 2021) |
| *Aurantiochytrium* sp. | 25 °C | One-step fermentation | DHA | / | / | (Wang et al., 2022) |
| *Aurantiochytrium* sp. SZU445 | 5 °C,15°C | Staged temperature control method | PUFAs | 3-ketoacyl-CoA synthase, ketoreductase, rfbB, ELO, FAS ↑ | PA, PC, PE, PG, and PI ↑ | This study |
