# Supplementary material for: Comparative transcriptomic and lipidomic analyses indicate that cold stress enhanced the production of the long C18–C22 polyunsaturated fatty acids in Aurantiochytrium sp
Source: Front Microbiol. 2022 Sep 20;13:915773. doi: 10.3389/fmicb.2022.915773 (PMC9530390; doi:10.3389/fmicb.2022.915773)
Supplement: Supplementary file 2 [file Table_2.docx]

**Table S2 The Gene sequence of internal standard for qRT-PCR analysis**

| **Gene ID** | **Sequence** |
| --- | --- |
| GME13964_g | ATGCAGATCCTAAACACTGTCGTGGTCCTCGCCTCCGTGGTGGCCAGCGCCAATGCGTTCTACCTTCCGGGTGTGGCCCCTCATGAATATAAGTCTGGTG  AACAGGTGGCTCTCAAGGTTAACGCCATGACAAGCATGCGCACCCACCTTGACTACAGCTTCTACTCCCTGCCACACTGCCTTCCCGAGGGTGGCGTTAA  AGACGCTCCCGAAAACCTTGGTGAGCACCTCACCGGCAACCTTGTGCAGAACTCCCCGTACGATATCAGAATGTATCAGGAGGAGAAGTGTCCCAAGGTT  CTCTGTACCAAGGAGTACACCGCCAAGGAGGTTGGTCAGTTCGTAGAGAAGATCGAGGAACAATACCTCGTGAACTGGATTGTGGACAACCTTCCCGTTG  CCTACCGCACCCTCAATGAGGCCAAGACCAAGCAGCTCAGATACTACTCTCACGGCTTTCCCCTTGGTGGCATGATGGTCTCGCCCTCAAACCCGAGTGA  GTCCCACTACTTCCTCAACAACCACGTTAACATTATCCTCCAGTACCATGTGCCTGACTTTGAGGTAGCTGCCAACCAGGACAAGATCCAGTTTGAGGAG  CTCCCTGGGCGCATTGTCGGAGCATTCGCAGAACCTTACTCCGTCAAGCACAACCTTGCGAGCGATGGAAGCATCAAGGAGCAGTACGAGTGTGACTTCG  CCAACGCAGAGGCCAAGGACACCAAGTACGGTCTCATTCTCGACGAGAAGAAGGCCAAGACCACCGTTGTCTGGTCTTACAGCGTCATGTGGGAGAAGTC  AAACATCAAGTGGGCCTCTCGCTGGGACGTTTACCTTACCTCCAAGAACAAGCGCAGTGACGAGGTGCACTGGTTCTCCATTATCAACTCTCTCCTCATC  GACGTCTTCCTCACCGGTATGGTCGCAATGATCCTTATGCGCACTGTCTACCGTGACCTCTCCCGCTACAACCGCGTGCCCACCGAGGAGGAGCGCATGG  AGGAGCGTGATGAGAGCGGTTGGAAGCTTGTCCACGGAGATGTCCTGCGCCCGCCCACTGCCCCTATGCTCTTTGCAGTCACTGTTGGTACCGGTATCCA  AGTTCTTGGTATGGCCGTTGCAACTATTGTCTTTGCTGCTGTCGGATTCCTCAGCCCTGCTTACCGTGGCTCTCTCATGACCGGTCTCCTGCTGCTCTTT |
| GME9330_g | ATGCAGATCCTAAACACTGTCGTGGTCCTCGCCTCCGTGGTGGCCAGCGCCAATGCGTTCTACCTTCCGGGTGTGGCCCCTCATGAATATAAGTCTGGTG  AACAGGTGGCTCTCAAGGTTAACGCCATGACAAGCATGCGCACCCACCTTGACTACAGCTTCTACTCCCTGCCACACTGCCTTCCCGAGGGTGGCGTTAA  AGACGCTCCCGAAAACCTTGGTGAGCACCTCACCGGCAACCTTGTGCAGAACTCCCCGTACGATATCAGAATGTATCAGGAGGAGAAGTGTCCCAAGGTT  CTCTGTACCAAGGAGTACACCGCCAAGGAGGTTGGTCAGTTCGTAGAGAAGATCGAGGAACAATACCTCGTGAACTGGATTGTGGACAACCTTCCCGTTG  CCTACCGCACCCTCAATGAGGCCAAGACCAAGCAGCTCAGATACTACTCTCACGGCTTTCCCCTTGGTGGCATGATGGTCTCGCCCTCAAACCCGAGTGA  GTCCCACTACTTCCTCAACAACCACGTTAACATTATCCTCCAGTACCATGTGCCTGACTTTGAGGTAGCTGCCAACCAGGACAAGATCCAGTTTGAGGAG  CTCCCTGGGCGCATTGTCGGAGCATTCGCAGAACCTTACTCCGTCAAGCACAACCTTGCGAGCGATGGAAGCATCAAGGAGCAGTACGAGTGTGACTTCG  CCAACGCAGAGGCCAAGGACACCAAGTACGGTCTCATTCTCGACGAGAAGAAGGCCAAGACCACCGTTGTCTGGTCTTACAGCGTCATGTGGGAGAAGTC  AAACATCAAGTGGGCCTCTCGCTGGGACGTTTACCTTACCTCCAAGAACAAGCGCAGTGACGAGGTGCACTGGTTCTCCATTATCAACTCTCTCCTCATC  GACGTCTTCCTCACCGGTATGGTCGCAATGATCCTTATGCGCACTGTCTACCGTGACCTCTCCCGCTACAACCGCGTGCCCACCGAGGAGGAGCGCATGG  AGGAGCGTGATGAGAGCGGTTGGAAGCTTGTCCACGGAGATGTCCTGCGCCCGCCCACTGCCCCTATGCTCTTTGCAGTCACTGTTGGTACCGGTATCCA  AGTTCTTGGTATGGCCGTTGCAACTATTGTCTTTGCTGCTGTCGGATTCCTCAGCCCTGCTTACCGTGGCTCTCTCATGACCGGTCTCCTGCTGCTCTTT  ATCTTCATGGGTATTGCTGCAGGATACTTCTCTGCTCGTACCTACAAGGAGTTCGCCGGCCTTGAGTGGCAGCGTTGCACCCTTCTCACTGCCACTTTGT  ACCCTGGTATCATCAGCGCTGTGCTCTTCCTCCTTAACCTCGTGGTTTGGGCCGAGGGATCCACCACCGCCGTGCCCTTCGGTTCCATCGTGGCTGTGCT  TCTGCTCTACTTCTGCATCAGCGTGCCTCTCACCTTCTTCGGTGCCTACCTTGGCTACAAGAAGGACCTCGAGAAGCCGCCCGTCATCACCCAGGACATC  CCGCGTGCCATCCCCGAGCAACCCTGGTTCATGAACCCTGTGCTCACTGTGCTCATTGGTGGCATCCTTCCTTTTGGTGCTATCTTTGTCGAGCTCTTCT  TCGTTTTGTCCTCTCTTTGGCTTGACCAGTTCTTCAGTCTCTTTGGCTTCCTCTTCATTGTCTTTATCATCCTCATTATCACATGCGCTGAGATCACCGT  TGTGCTTACCTACTTTGCGCTCTGTGGTGAGGACTACCGCTGGCGCTGGCGTGCCTTCTTTACTTCGGGTTCGTCTGCTCTCTACCTCTTCCTCTACAGT  GTCTTCTACTTCTCCAGCCGCCTCAAGATGGACTTGATGGTCAGTGCCATCCTCTACTTCGGCTACATGACTATTTTCTCGCTCCTCTTCTTCCTTGCAA  CTGGCGTTGTCGGCTACTACGCCTCCCGCAAGTTTATCTACAAGATCTACGAGCTCGTCAAGCTCGACTAA |
