# Supplementary material for: Comparative transcriptomic and lipidomic analyses indicate that cold stress enhanced the production of the long C18–C22 polyunsaturated fatty acids in Aurantiochytrium sp
Source: Front Microbiol. 2022 Sep 20;13:915773. doi: 10.3389/fmicb.2022.915773 (PMC9530390; doi:10.3389/fmicb.2022.915773)
Supplement: Supplementary file 3 [file Table_3.docx]

**Table S3 The Primer sequence of DEGs for qRT-PCR analysis**

| **Gene ID** | **Sequences of primers (5’ to 3’)** |
| --- | --- |
| GME4589-F | CTGGCTCCGCTCTCGAGTCT |
| GME4589-R | CCTCGGTGGCCTTCTCAGAA |
| GME11161-F | ATGAAGCTGTACGCTCGCCC |
| GME11161-R | TGACGTCGCTCTCCTTGACG |
| GME5172-F | AGATTGTCCAGGAGGAGCGC |
| GME5172-R | GAGACCCTGCTCGACCTGGT |
| GME3841-F | CTCTCTGCTTCGTGGCCGTA |
| GME3841-R | GGAGGACATTGTGCTCAGCG |
| GME32-F | CAATGGCGTTGAGTTCGAGG |
| GME32-R | CGTCGATCTGCATCAAAGGC |
| GME1043-F | CTTCTGATGCCGTTGAGCGT |
| GME1043-R | CAGCTTTGGCGGTATCCATG |
| GME4704-F | AGGATCACCACGTGCACCAT |
| GME4704-R | CAAAGCCCCGGAAGGACTTA |
| GME10254-F | CCAAGGCGAGAACGATGTTG |
| GME10254-R | ATTCCGGATCCACCACCAGT |
| GME7548-F | AGCAGCCCCTCAAGGTTGTC |
| GME7548-R | GGTGTAAGCATCGGTGTGGC |
| GME34-F | CGTTCAATTTGTTCCGGCTG |
| GME34-R | ACTTGCTGTACCACTCGGCG |
| GME403-F | CGTCAGCATTGCCGGTTACT |
| GME403-R | CCGTTGCCATTGGTGTTGTT |
