# Supplementary material for: Comparative transcriptomic and lipidomic analyses indicate that cold stress enhanced the production of the long C18–C22 polyunsaturated fatty acids in Aurantiochytrium sp
Source: Front Microbiol. 2022 Sep 20;13:915773. doi: 10.3389/fmicb.2022.915773 (PMC9530390; doi:10.3389/fmicb.2022.915773)
Supplement: Supplementary file 4 [file Table_4.docx]

**Table S4 The quality of RNA**

| **Sample name** | **Concentration**  **(ng/μg)** | **Volume**  **(μL)** | **Total quantity**  **(μg)** | **OD260/280** | **OD260/230** | **RIN** | **28S/18S** |
| --- | --- | --- | --- | --- | --- | --- | --- |
| **Th25_1** | 1690 | 40 | 67.6 | 2.12 | 2.16 | 9.4 | 1.5 |
| **Th25_2** | 1448 | 40 | 57.92 | 2.14 | 1.43 | 9.4 | 1.6 |
| **Th25_3** | 1538 | 40 | 61.52 | 2.14 | 1.43 | 9.8 | 1.7 |
| **Th15_1** | 1440 | 40 | 57.6 | 2.18 | 2.09 | 9.8 | 1.8 |
| **Th15_2** | 1430 | 40 | 57.2 | 2.19 | 2.06 | 9.7 | 1.8 |
| **Th15_3** | 1480 | 40 | 59.2 | 2.2 | 2.02 | 9.3 | 1.7 |
| **Th5_1** | 1740 | 40 | 69.6 | 2.13 | 2.2 | 9.5 | 1.5 |
| **Th5_2** | 1700 | 40 | 68 | 2.14 | 2.04 | 9.2 | 1.8 |
| **Th5_3** | 1780 | 40 | 71.2 | 2.2 | 2.15 | 9.4 | 1.7 |
