# Supplementary figures and images for: Comparative transcriptomic and lipidomic analyses indicate that cold stress enhanced the production of the long C18–C22 polyunsaturated fatty acids in Aurantiochytrium sp
Source: Front Microbiol. 2022 Sep 20;13:915773. doi: 10.3389/fmicb.2022.915773 (PMC9530390; doi:10.3389/fmicb.2022.915773)

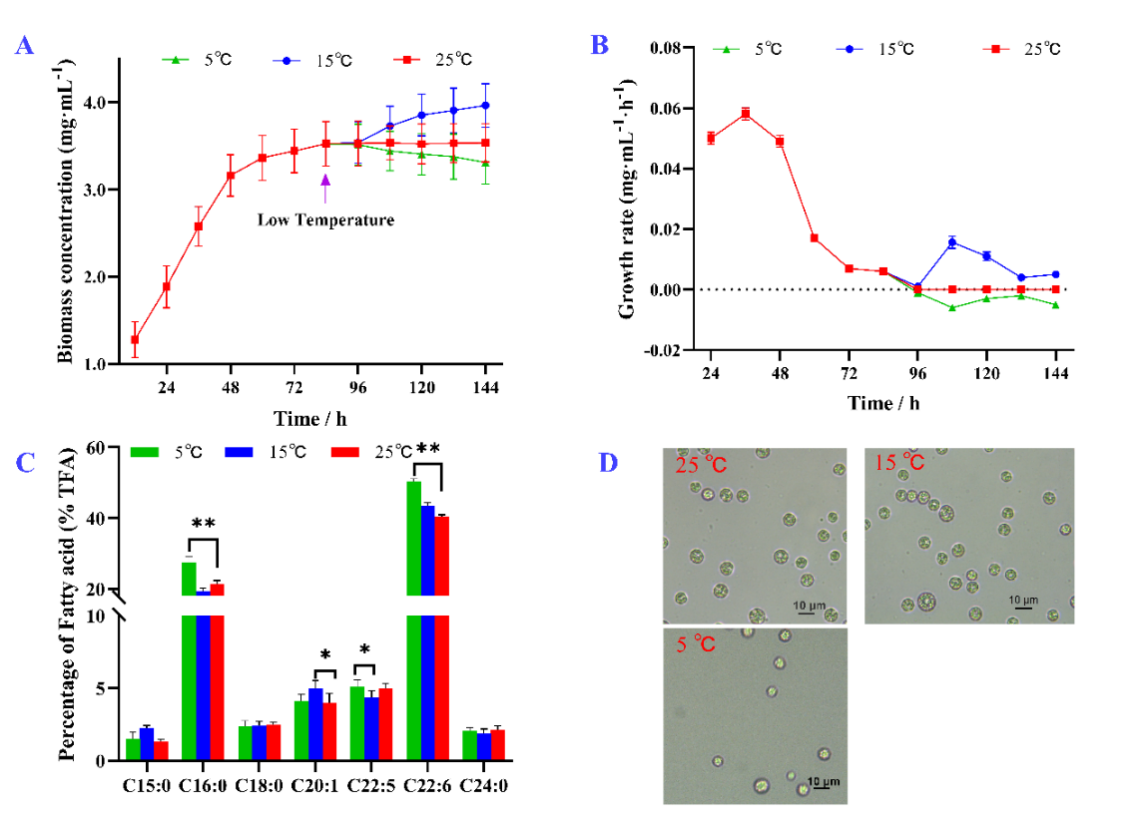

Supplement: Supplementary file 5 [file Image_1.png]

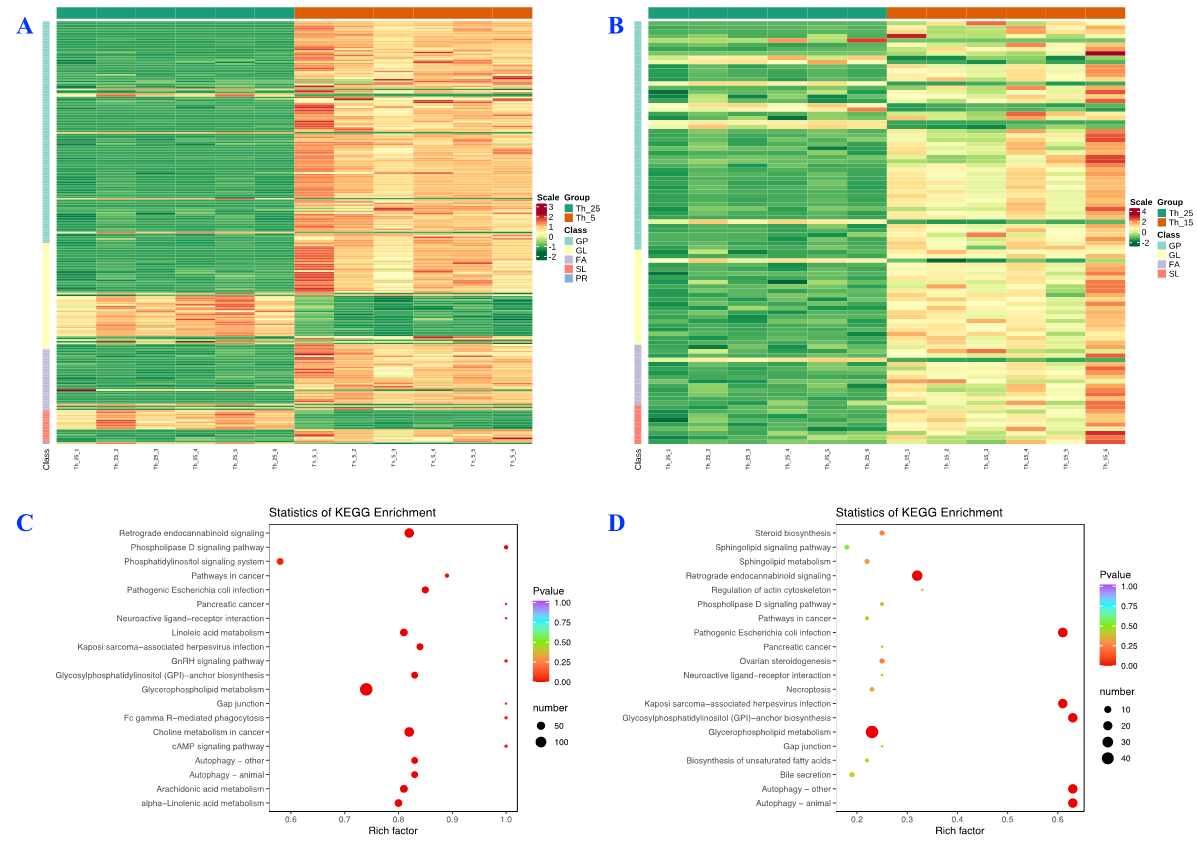

Supplement: Supplementary file 6 [file Image_2.png]

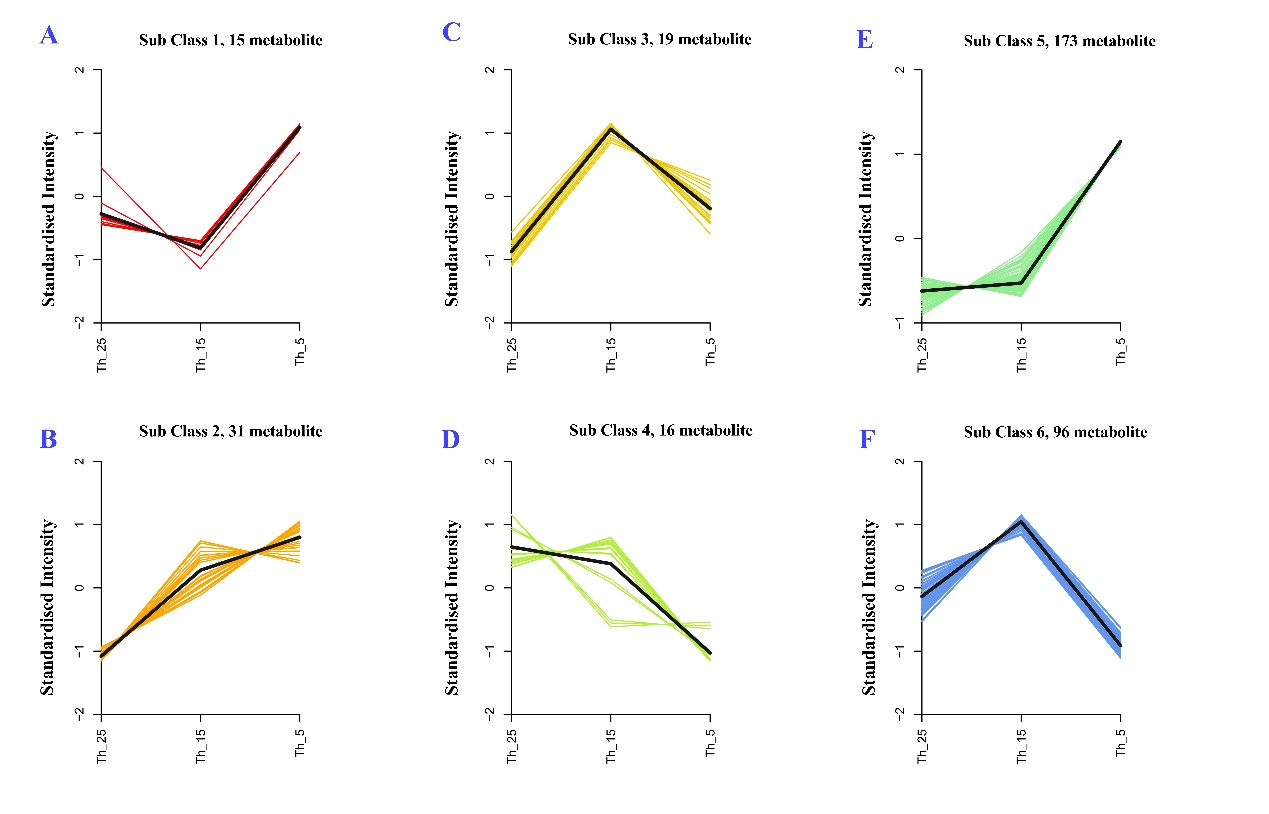

Supplement: Supplementary file 7 [file Image_3.png]

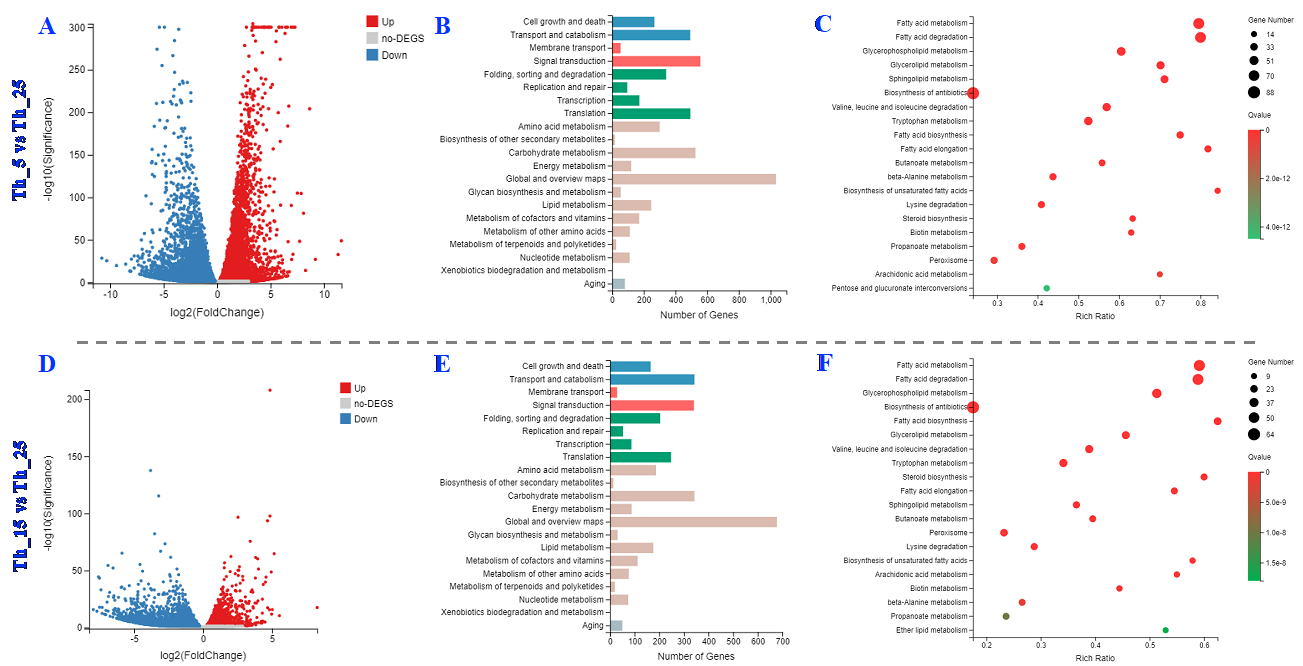

Supplement: Supplementary file 8 [file Image_4.png]

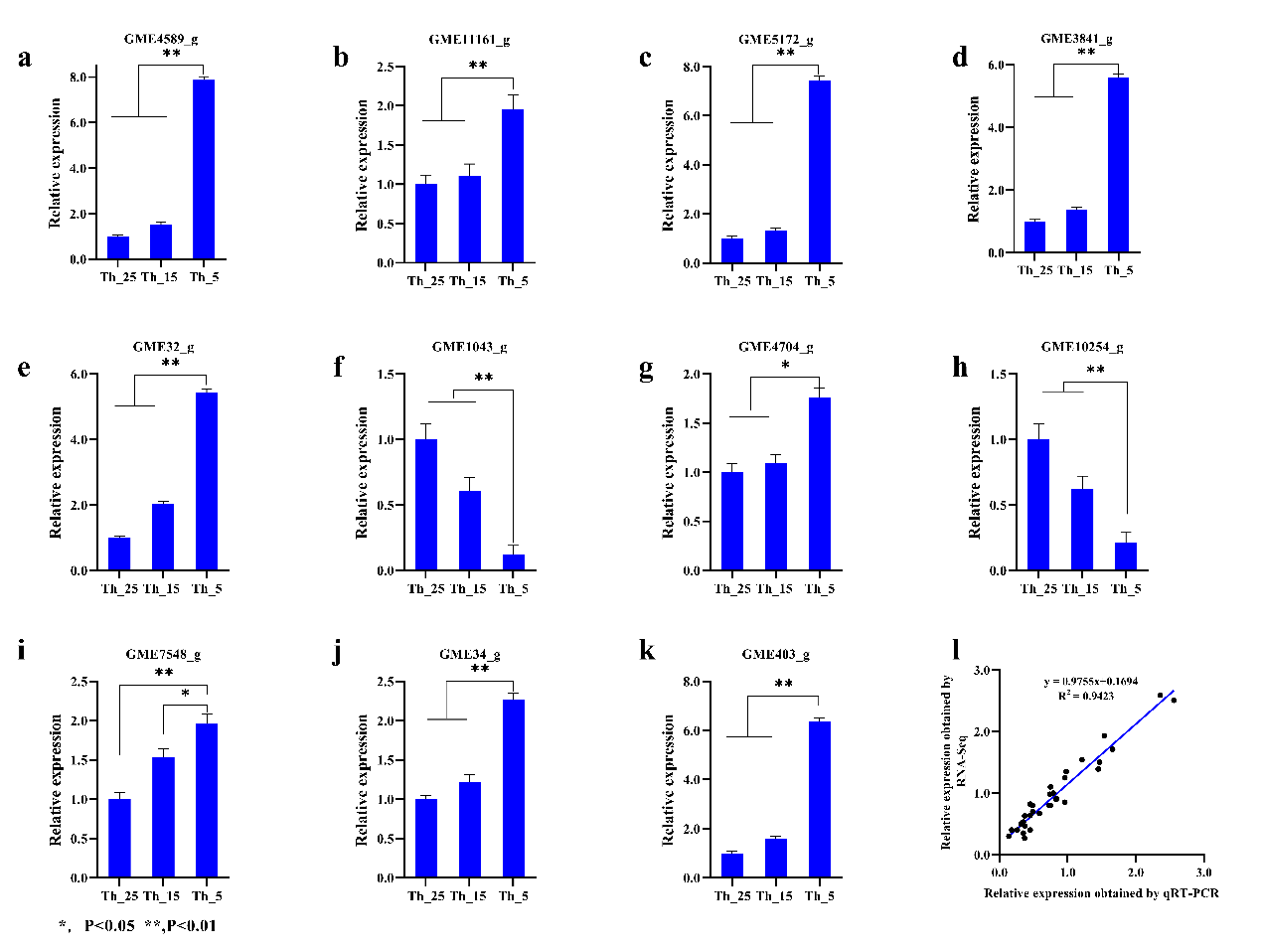

Supplement: Supplementary file 9 [file Image_5.png]
